# Study on the influence of rock bridge angle on the size effect of rock uniaxial compression

**DOI:** 10.1371/journal.pone.0299230

**Published:** 2024-05-24

**Authors:** Gaojian Hu, Zhonghua Xie, Yuan Xing, Yangyong Gu, Haoran Feng, Lei Sun

**Affiliations:** 1 School of Civil Engineering, Shaoxing University, Shaoxing, Zhejiang, China; 2 Huahui Engineering Design Group Co., Ltd., Shaoxing, Zhejiang, China; 3 School of Energy Science and Engineering, Henan Polytechnic University, Jiaozuo, Henan, China; 4 Shaoxing Daming Electric Power Construction Co., Ltd., Shaoxing, Zhejiang, China; 5 Shaoxing Power Supply Company, State Grid Zhejiang Electric Power Co., Ltd., Shaoxing, Zhejiang, China; 6 Department of Intelligent Manufacturing, Yantai Vocational College, Yantai, Shandong, China; Xi’an University of Science and Technology, CHINA

## Abstract

As a basic parameter of rock, the rock bridge angle plays an important role in maintaining the stability of rock masses. To study the size effect of rock bridge angle on the uniaxial compressive strength of rocks, this paper adopts the principle of regression analysis and combines numerical simulation to carry out relevant research. The research results indicate that: (1) the uniaxial compressive strength decreases with the increase of the rock bridge angle, showing a power function relationship; (2) The uniaxial compressive strength decreases with the increase of rock size and tends to stabilize when the rock size is greater than 350 mm, showing a significant size effect. (3) The fluctuation coefficient of compressive strength increases with the increase of rock bridge angle and decreases with the increase of rock size; When the rock size is 350 mm, the fluctuation coefficient is less than 5%; (4) The characteristic compressive strength and characteristic size both increase with the increase of the rock bridge angle.

## 1. Introduction

As a discontinuous medium material, rock widely exists in nature. The special structure caused by defects such as pores, cracks, and joints inside the rock results in significant size effects. As one of the essential rock mechanics parameters, uniaxial compression strength (UCS) also has size effect. The rock bridge angle is a key indicator for evaluating rock stability and has a significant impact on the UCS. Therefore, it is of great reference value to study the influence of rock bridge angle and size effect on UCS, which can effectively prevent engineering accidents and provide essential guidance for engineering practice.

As one of the important indexes to evaluate the stability of rock, the rock bridge angle significantly influences the UCS. Scholars have studied it from rock bridge length and crack development perspectives. Xiong et al. [1] found that the rock bridge angle significantly affects the UCS when the length of the rock bridge is constant. Yang et al. [2] studied the influence of rock bridge angle on UCS from the perspective of fracture development and found that the peak strength of rock depends on the rock bridge angle. Jiang et al. [3] used 3D printing technology to establish a double crack model and obtained the failure characteristics of the rock. Scholars also studied it from the perspectives of water content, crack development, and joint dip angle. Yu et al. [4] studied the relationship between the rock bridge angle and UCS under different water content by using photographic monitoring technology, and they found that the influence of the rock bridge angle on the UCS gradually decreased with the increase of water content. Tian et al. [5] studied from the perspective of fracture evolution and found that the UCS obtained the minimum and maximum values when the rock bridge angle was 60° and 120°, respectively. Chen et al. [6] explored from the perspective of fracture evolution characteristics and shows that with the increase of rock bridge angle, the UCS shows a trend of decreasing and then increasing. Based on the sliding crack theory, Sun et al. [7] explored from the perspective of joint dip angle and found that the UCS reached the minimum when the rock bridge angle was 45°. Scholars have also established the relationship between the rock bridge angle and UCS. Li et al. [8] explored the relationship between the rock bridge angle and UCS under dynamic loading conditions by conducting dynamic impact tests on marble specimens and established the connection. Wang et al. [9] investigated the failure mechanism of rocks using a new sealed testing platform. From these literatures, we can find that the rock bridge angle is closely related to the UCS, but the influence of size effect is not considered, and the relationship between UCS and rock bridge angle is not established.

The UCS has a size effect. As the size increases, the UCS will gradually decrease until it tends to be stable. Scholars have studied it from the aspects of loading conditions, ratio of height to diameter, water content, rock type, and strain rate. For example, Zhou et al. [10] used an RMT testing machine to study the relationship between rock size and UCS under quasi-static and dynamic conditions, and accurately derived their patterns by improving the Weibull distribution formula. Fu et al. [11] used granite and limestone for laboratory uniaxial compression test and found that the height-diameter ratio had a significant effect on UCS. Wang et al. [12] explored from the perspective of moisture content and found that in the dry and saturated state, the UCS decreases slightly with increasing volume. Wang et al. [13] used a large rigid test device to study the UCS from the perspective of aspect ratio, and found that as the aspect ratio increased, the UCS showed a trend of first decreasing and then increasing. Zhou et al. [14] studied from the perspective of strain rate and established the theory of concrete strength size effect related to strain rate. Zhou et al. [15] proposed a scale model containing strain rate based on fractal theory and dynamic fracture mechanics. The above results show that the UCS is negatively correlated with size, but the relationship between UCS and size has not been established. In the study of size effect, there are relatively few studies considering the rock bridge angle, and the influence of the rock bridge angle on the size effect of the UCS has not been obtained.

When the rock mechanics parameters no longer change with rock size, this critical size is called the characteristic size. Scholars have studied it from the aspects of deformation features, size prediction, permeability, energy consumption density, joint parameters, rock roughness and parallel joints. For example, Chen et al.[16] explored the characteristic size of rock from the perspective of spatial structure of rock mass and discussed the correlation between geometric and mechanical size effect of rock mass. Bamford T et al. [17] used deep neural network (DNN) model to predict the characteristic size and found that the accuracy of DNN model prediction and manual labeling prediction was similar. Yang et al. [18] studied from the perspective of permeability and found that when the rock size is close to the characteristic size, the permeability of the rock becomes stable and isotropic. Based on continuous discontinuous element method (CDEM), Feng et al. [19] explored from the perspective of roughness and established a mathematical relationship between roughness and characteristic size. Hu et al. [20] studied the influence of joint roughness on compressive strength and established a mathematical model of roughness and characteristic size. Hu et al. [21] explored the influence of joint spacing on compressive strength and obtained the specific relationship between joint spacing and characteristic size. The above scholars have explored its relationship with characteristic size from the perspective of deformation, permeability, and energy consumption, but have not established its mathematical relationship, nor have they considered the influence of rock bridge angle.

In this paper, the influence of rock bridge angle and rock size on UCS is explored. The relationship between UCS and rock bridge angle and the relationship between UCS and rock size are quantitatively described. The relationship between rock characteristic size and characteristic strength and rock bridge angle has been studied.

## 2. Establishment of numerical model

### 2.1 Numerical simulation schemes

**Fig 1:**
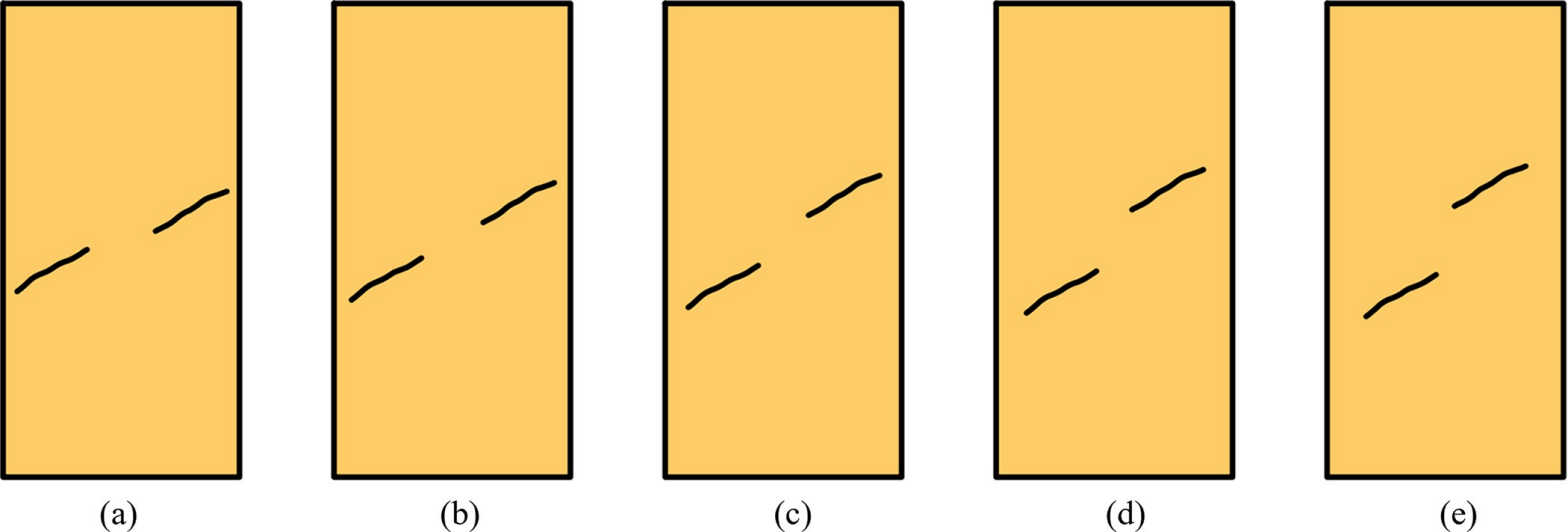
Calculation models of Scheme 1 (a)15°, (b)30°, (c)45°, (d)60°, (e)75°.

**Fig 2:**
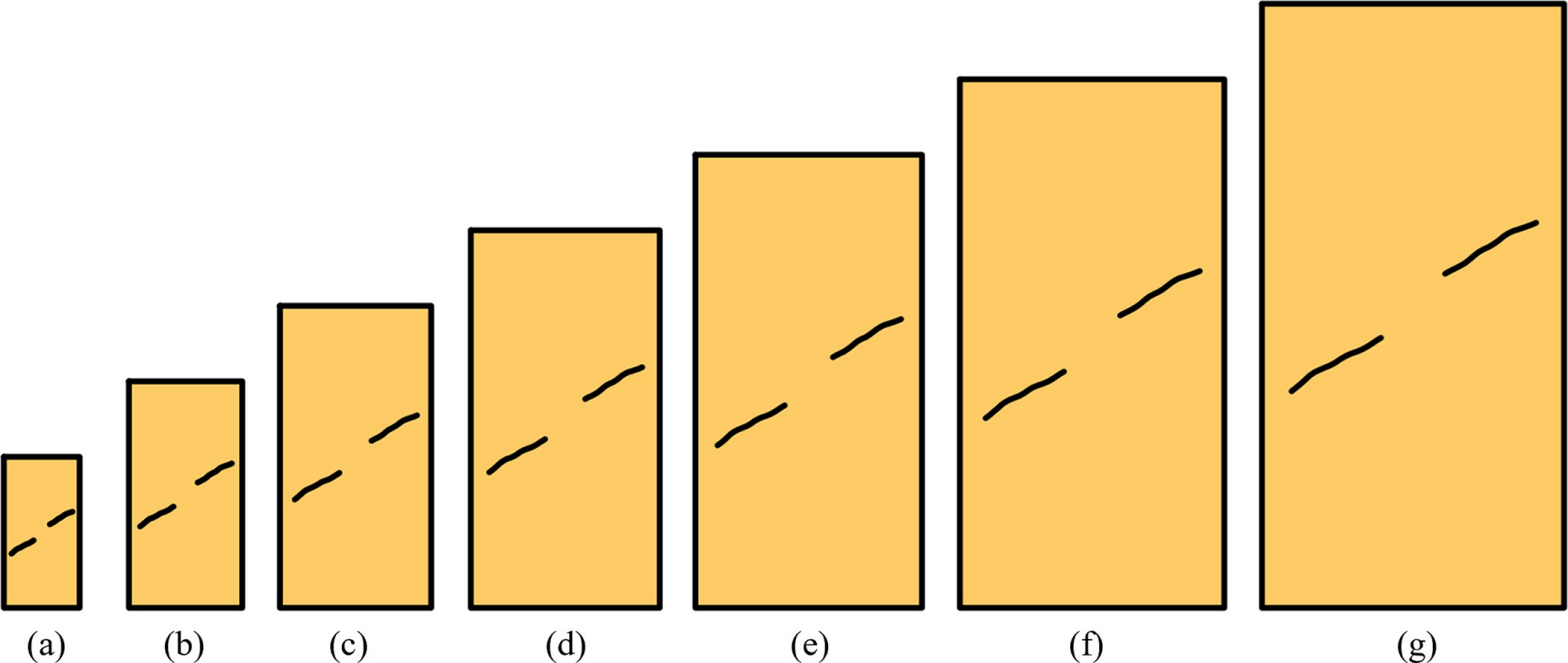
Calculation models of Scheme 10 (a) 100 mm, (b) 150 mm, (c) 200 mm, (d) 250 mm, (e) 300 mm, (f) 350 mm, (d) 400 mm.

The numerical simulation program in this paper includes two aspects. Research content 1 is about the relationship between the rock bridge angle and the UCS, the rock bridge angles are 15°, 30°, 45°, 60°, and 75°, including schemes 1–7, and the content of Scheme 1 is shown in Fig 1. Research content 2 is about the relationship between rock size and UCS, the rock sizes are 100 mm, 150 mm, 200 mm, 250 mm, 300 mm, 350 mm, and 400 mm, including schemes 8–12, and the content of Scheme 10 is shown in Fig 2. The specific schemes are shown in Table 1, where size refers to the short side size of the numerical model.

**Table 1:** Combination table of rock size and rock bridge angle.

Simulation program	Size/mm	Scheme 8	Scheme 9	Scheme 10	Scheme 11	Scheme 12
		15°	30°	45°	60°	75°
Scheme 1	100	100×15°	100×30°	100×45°	100×60°	100×75°
Scheme 2	150	150×15°	150×30°	150×45°	150×60°	150×75°
Scheme 3	200	200×15°	200×30°	200×45°	200×60°	200×75°
Scheme 4	250	250×15°	250×30°	250×45°	250×60°	250×75°
Scheme 5	300	300×15°	300×30°	300×45°	300×60°	300×75°
Scheme 6	350	350×15°	350×30°	350×45°	350×60°	350×75°
Scheme 7	400	400×15°	400×30°	400×45°	400×60°	400×75°

### 2.2 Selection of rock mechanics parameters

#### 2.2.1 The rock mechanical parameters

RFPA software is a numerical simulation tool based on rock mechanics theory, which can simulate the overall failure and crack propagation process of rock [22]. Based on the elastic damage theory of rock mechanics and the modified Coulomb failure criterion, the software combines the three-dimensional heterogeneity of materials and the randomness of defect distribution and analyzes and simulates the structural characteristics of rocks by means of numerical methods. One of the main features of RFPA software is that it can simulate the crack propagation process of rock, including cracking zone, fault, and nonlinear elasticity. Secondly, the software also provides different boundary and constraint conditions and anisotropic parameters of rock to help researchers reproduce the experimental process more realistically. In addition, it also supports a variety of element types, which can easily realize the simulation of different rock structures.

In this simulation experiment, the UCS of the rock is 101.34 MPa, the elastic modulus is 4874 MPa, the internal friction angle is 48.32°, and the Poisson’s ratio is 0.25. The UCS of the joint is 0.01 MPa, the elastic modulus is 0.01 MPa, the internal friction angle is 40°, the Poisson’s ratio is 0.25, and the roughness is 8.19. The parameters are shown in Table 2.

**Table 2:** Mechanical parameters.

Material	UCS/MPa	Elastic modulus/MPa	Poisson ratio	Internal friction/°
Rock	101.34	4874	0.25	48.32
Joint	0.01	0.01	0.25	40

The process of obtaining crack roughness is as follows: Firstly, the contour curve of the rock structural plane on the mine slope is drawn using a contour curve instrument. Then, data extraction of the contour curve is carried out using a scanner and MATLAB software, and it is converted into CAD curves. Finally, the CAD curves are imported into RFPA software for simulation research.

#### 2.2.2 Boundary conditions

**Fig 3:**
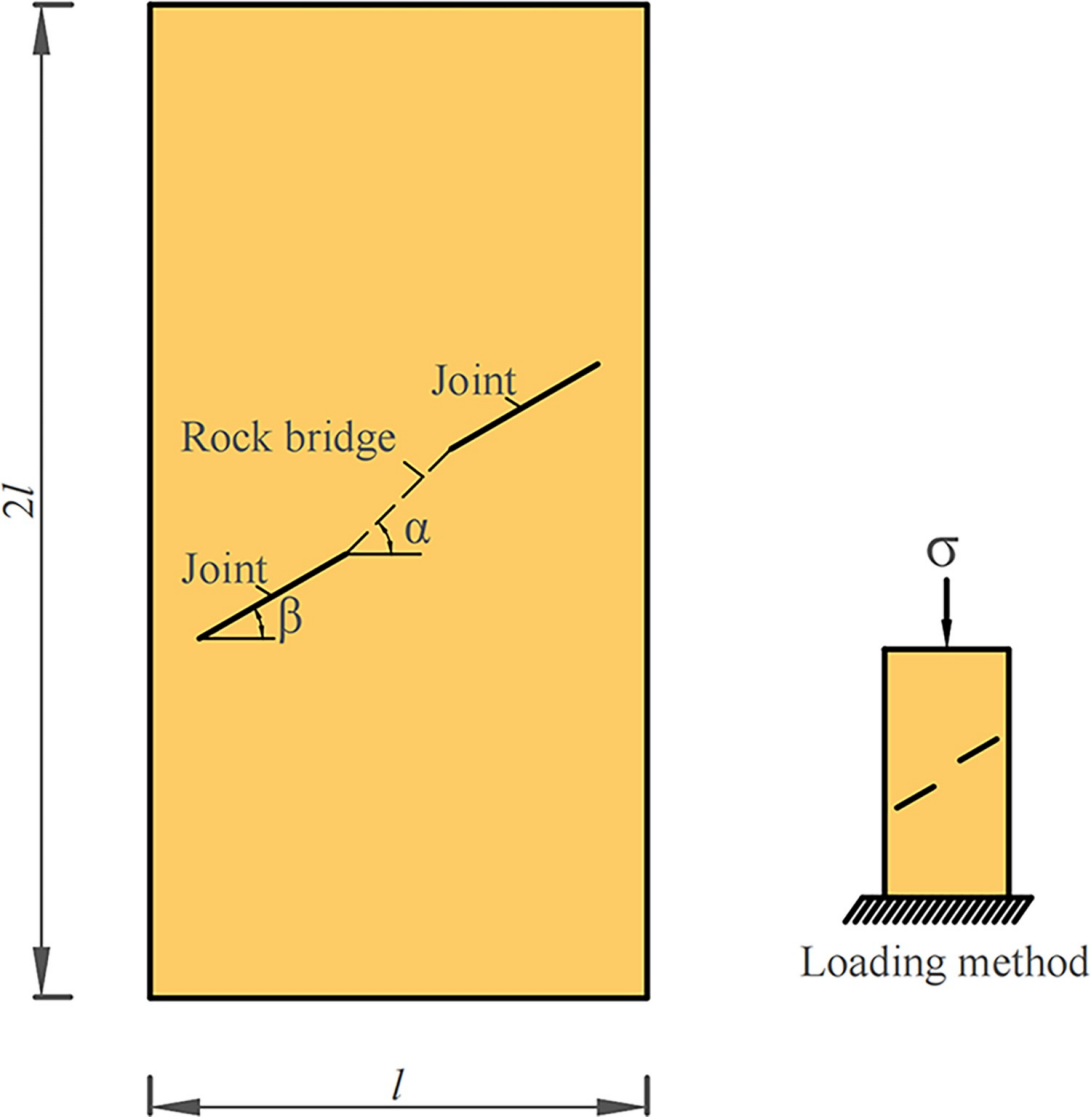
Numerical simulation model.

The model is shown in Fig 3, with a ratio of 2:1 between the long and short edges. In the model α is the rock bridge angle, β is joint inclination angle. The constraint conditions of the model are that both sides are free boundaries, the lower end is fixed, and the upper part bears the load. During the simulation process, displacement loading was used, with an initial loading amount of 0 and a displacement amount of 0.01mm. The rock was loaded until the specimen was destroyed, and the material used was tuff.

## 3. Analysis of research results

### 3.1 Study on the relationship between rock bridge angle and UCS

#### 3.1.1 Stress—strain curve analysis

**Fig 4:**
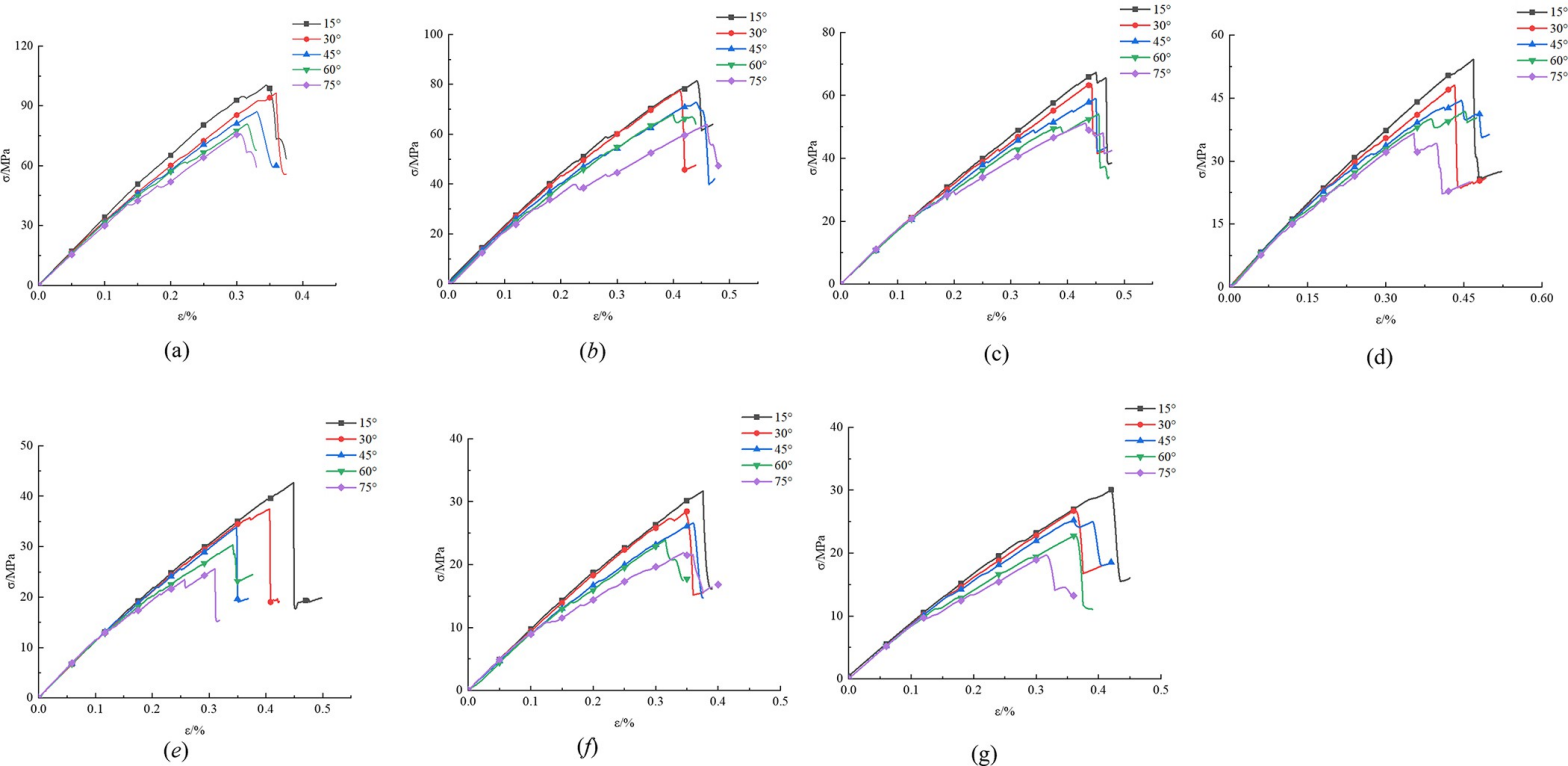
Stress-strain curves under different rock bridge angle (a) 100 mm, (b) 150 mm, (c) 200 mm, (d) 250 mm, (e) 300 mm, (f) 350 mm, (g) 400 mm.

According to the research content 1, Fig 4 gives the stress-strain curves of different rock bridge angles when the rock size is 100 mm, 150 mm, 200 mm, 250 mm, 300 mm, 350 mm, and 400 mm.

According to Fig 4, the UCS of rock under each scheme is shown in Table 3.

**Table 3:** UCS under different rock bridge angle.

Simulation program	Size/mm	UCS /MPa				
		Scheme 8	Scheme 9	Scheme 10	Scheme 11	Scheme 12
		15°	30°	45°	60°	75°
Scheme 1	100	100.44	96.43	88.25	82.08	76.94
Scheme 2	150	83.51	78.27	73.16	67.29	63.87
Scheme 3	200	67.28	63.46	58.88	54.03	51.96
Scheme 4	250	54.17	48.24	44.18	40.22	36.54
Scheme 5	300	42.63	37.44	33.84	30.34	25.56
Scheme 6	350	31.69	28.66	26.84	23.91	21.86
Scheme 7	400	30.24	27.14	25.26	23.25	19.91

As shown in Fig 4A, the stress of rock increases linearly with strain, and the deformation of rock is mainly elastic deformation, with the rock in the stage of elastic deformation. Subsequently, the stress nonlinearly increases to the peak strength with strain, and then unstable failure occurs in the strain range of 0.30% to 0.35%. The rock is in the stage of unstable fracture development. With the failure of the internal structure of the rock, the UCS rapidly decreases with the increase of strain, but does not decrease to zero, indicating that the rock still has a certain residual strength after fracture, and the rock is in the post fracture stage.

To explore the relationship between the rock bridge angle and UCS, the analysis is combined with Fig 4B and Table 3. The rock UCS corresponding to rock bridge angles of 15°, 30°, 45°, 60°, and 75° is 83.51 MPa, 78.27 MPa, 73.16 MPa, 67.29 MPa, and 63.87 MPa, respectively. The UCS decreases with the increase of the rock bridge angle. In the simulation, when the rock bridge angle is small, the rock has a stronger ability to resist failure, thus the compressive strength is higher. As the angle of the rock bridge becomes steeper, the ability of the rock to resist damage gradually decreases.

Through the analysis of Fig 4, we found that there is a relationship between rock size and its compressive strength. Specifically, when the rock size gradually increases, the compressive strength of the rock gradually decreases. When the rock size is 100 mm and 400 mm, the compressive strength reaches maximum and minimum values. In addition, we also found that when the rock size exceeds 350 mm, the UCS of different sizes changes little, showing a close trend. This indicates that when the rock size reaches a certain threshold, the size effect of rock gradually stabilizes, and the difference in UCS between rocks of different sizes is relatively tiny. The reason for this phenomenon may be related to the internal structure of the rock. When the rock size is small, the internal voids are small, and the stress distribution between particles is relatively uniform, so the rock has a strong ability to resist pressure. When the rock size increases, the internal voids increase, the stress distribution between particles becomes uneven, and the ability of rock to resist pressure gradually weakens. However, when the size of the rock increases to a certain extent, due to the limitations of the internal structure, the trend of the UCS begins to slow down or even stabilize.

According to the comprehensive analysis, it can be concluded that there is a negative correlation between rock size, rock bridge angle and UCS.

#### 3.1.2 Curve fitting of rock bridge inclination and UCS

**Fig 5:**
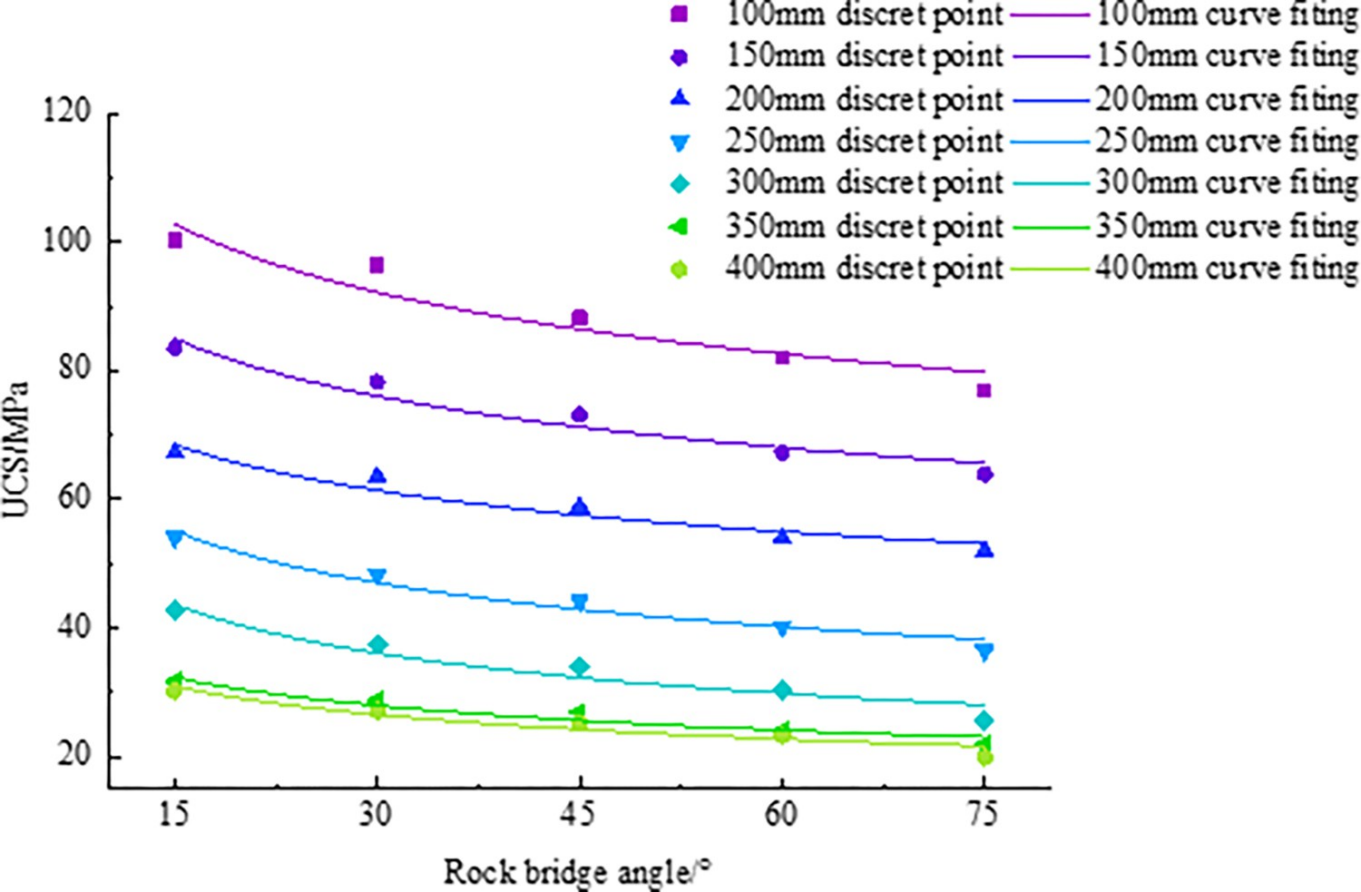
Curve fitting of UCS.

The data in Table 3 are further visualized, and the fitting curves of rock bridge angle and UCS are drawn, as shown in Fig 5. By analyzing the data using statistical methods, the linear regression equation is obtained, as shown in Table 4.

**Table 4:** Fitting relationship between rock UCS and rock bridge angle.

Rock size/mm	Fitting formula	R^2^
100	$\sigma (\alpha )=139.52\alpha^{-0.14}$	0.88
150	$\sigma (\alpha )=124.38\alpha^{-0.16}$	0.92
200	$\sigma (\alpha )=108.47\alpha^{-0.18}$	0.92
250	$\sigma (\alpha )=92.85\alpha^{-0.20}$	0.95
300	$\sigma (\alpha )=79.16\alpha^{-0.23}$	0.91
350	$\sigma (\alpha )=63.82\alpha^{-0.24}$	0.92
400	$\sigma (\alpha )=49.43\alpha^{-0.26}$	0.89

Where *σ*(*α*) is UCS, unit: MPa; *α* is the rock bridge angle, unit:°.

The analysis of Table 3 and Fig 5 shows that the UCS decreases gradually with the increase of the rock bridge angle. Further research shows that there are two poles in this trend for each size of rock, that is, the UCS when the rock bridge angle is 15° and 75°. When the rock size is 100 mm, it is found that when the rock bridge angle is 15°, the UCS is the maximum value, reaching 100.44 MPa, while when the rock bridge is 75°, the UCS is the minimum value, only 76.94 MPa, and the UCS decreases by 23.39%. By combining the analysis of Table 3 and Fig 5, we can better understand the properties of rocks and more accurately assess engineering risks. Based on the curves and its fitting relationships in Fig 5, we obtained the content in Table 4.

#### 3.1.3 Mathematical model of rock bridge angle and UCS

According to Table 4, we can saw that the rock bridge angle and UCS conform to the power function relationship, and the model is proposed as follows:

$$\sigma (\alpha )=a\alpha^{b}$$ (1)

Where *σ*(*α*) is the UCS, unit: MPa, *α* is the rock bridge angle, unit:°, *a* and *b* are regression parameters, which can be obtained by linear regression.

**Fig 6:**
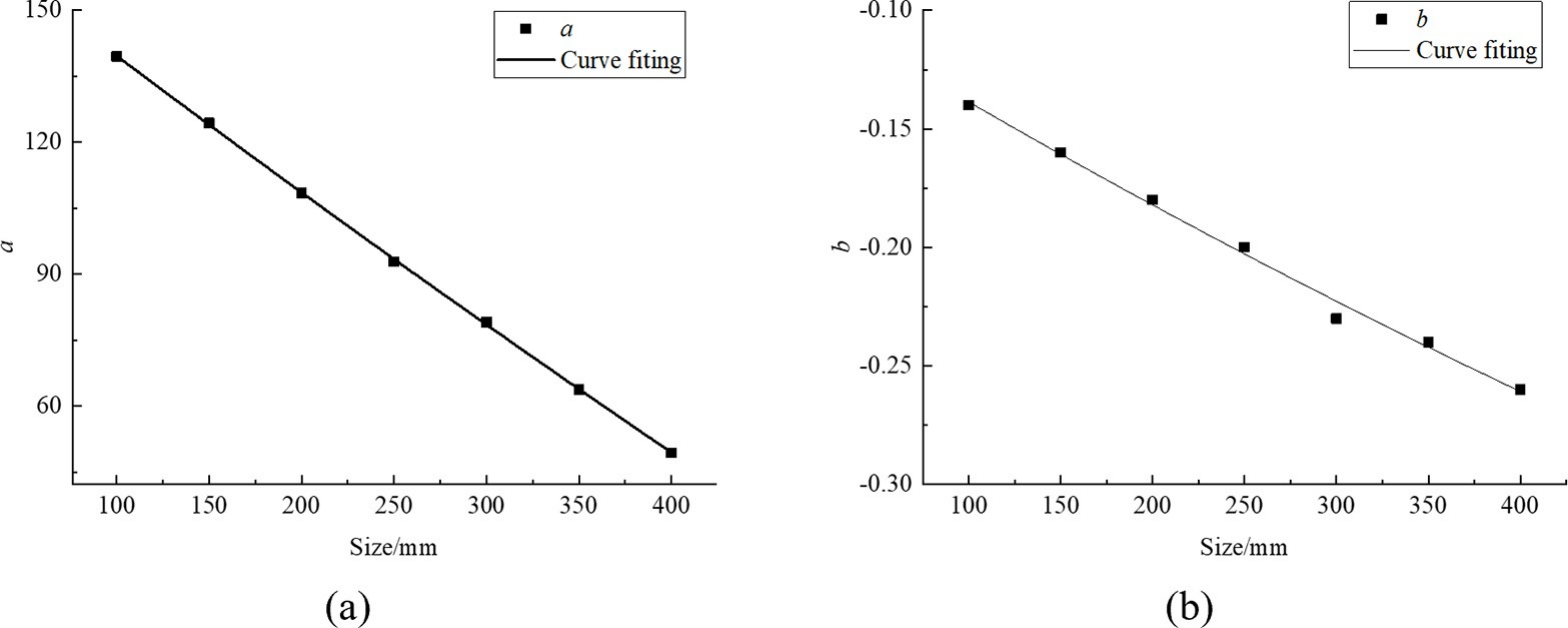
Fitting diagram of regression parameter (a) *a*, (b) *b*.

The parameters of regression equations were obtained by linear regression analysis of the data in Table 4. These regression parameters are organized in Table 5 to better understand the meaning and role of these parameters. At the same time, we further fit the regression parameters with different rock sizes into charts, as shown in Fig 6.

**Table 5:** Regression parameters under different rock sizes.

Size/mm Parameter	100	150	200	250	300	350	400
*a*	139.52	124.38	108.47	92.85	79.16	63.82	49.43
*b*	-0.14	-0.16	-0.18	-0.20	-0.23	-0.24	-0.26

According to Fig 6, the fitting error are all 0.99, and the relationship between parameters and rock size is sorted out as follows:

$$a=-611.56+983.31\times 0.99^{l}$$ (2)

$$b=-0.81+0.72\times 0.99^{l}$$ (3)

By incorporating the compiled relationship equations into Formula (1), the special relationship between the rock bridge angle and UCS is:

$$\sigma (\alpha )=(-611.56+983.31\times 0.99^{l})\alpha^{-0.81+0.72\times 0.99^{l}}$$ (4)

Where *σ*(*α*) is the UCS, unit: MPa, *α* is the rock bridge angle, unit:°, *l* is the rock size, unit: mm.

Eq (4) is applicable to the calculation of UCS under different rock bridge angles in rock slopes, but it is only applicable to rocks formed by two regular joints and has certain limitations.

### 3.2 Analysis of UCS fluctuation coefficient

The fluctuation coefficient is an important parameter in rock mechanics, which is closely related to the stability of rock mechanics parameters and plays an important role in judging the engineering properties of rock. Eq (5) gives the calculation formula of wave coefficient of rock [23]:

$$A_{l}=|\frac{k_{l}-{\bar{k}}_{l}}{{\bar{k}}_{l}}|$$ (5)

Among them, A_*l*_ is the fluctuation coefficient of UCS, k_*i*_ is the value of mechanical parameters when the model size is *l*. ${\bar{k}}_{l}$ is the average UCS of rock when the model size is greater than or equal to *l*. The smaller the fluctuation coefficient A_*l*_ is, the smaller the amplitude of change in UCS and the more stable it becomes when the model size is greater than or equal to l.

The UCS fluctuation coefficient calculated according to Eq (5) has been summarized in Table 6. This paper draws the relationship between the UCS fluctuation coefficient and rock size and rock bridge angle, as shown in Fig 8.

**Table 6:** Fluctuation coefficient of UCS.

angle° Size/mm	15	30	45	60	75
100	71.50	77.80	76.29	78.92	81.56
150	61.88	65.82	67.44	68.90	74.43
200	48.84	54.83	55.77	57.29	66.72
250	36.51	36.39	35.81	36.66	40.71
300	22.31	20.46	18.13	17.45	13.89
350	2.34	2.72	3.03	1.40	4.67

**Fig 7:**
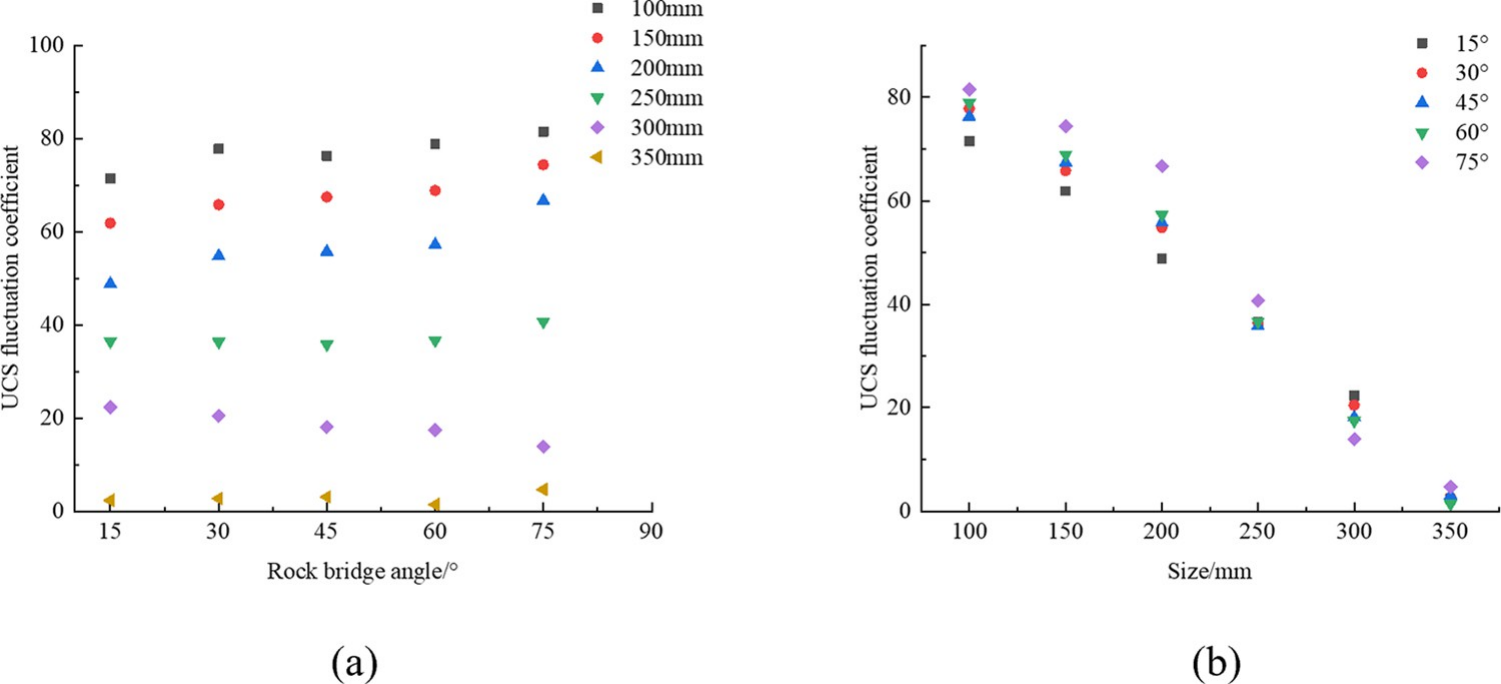
Variation of UCS fluctuation coefficient (a) Relationship with rock size, (b) Relationship with rock bridge angle.

Fig 7A shows the relationship between rock size and UCS fluctuation coefficient under different rock bridge angles. Combined with Table 6 and Fig 7A, taking the case of rock bridge angle of 15° as an example, it can be found that as the rock size increases from 100 mm to 350 mm, the fluctuation coefficient of UCS shows a significant downward trend, with a decrease of 96.73%. Specifically, the fluctuation coefficient decreased from 71.49% to 2.34%. This result indicates that as the rock size increases, the fluctuation coefficient of UCS will gradually decrease, especially when the rock size is 350 mm, the fluctuation coefficient is less than 5%, indicating that the mechanical parameters of the rock have stabilized. This conclusion can provide guidance for practical engineering applications and optimize the selection of rock size to improve the reliability and safety of engineering construction.

**Fig 8:**
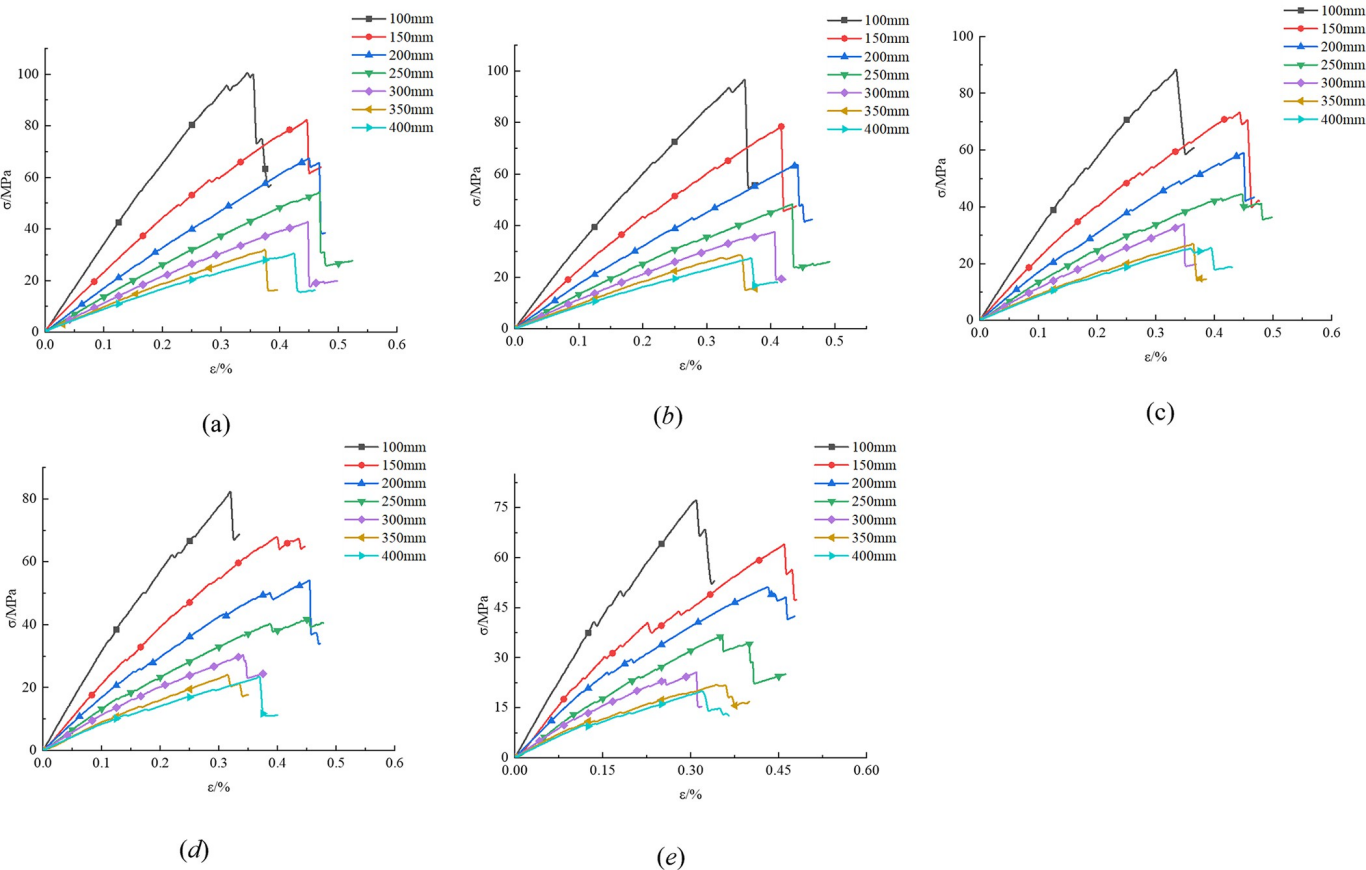
Stress-strain curves of rock under different rock sizes. (a)15°, (b) 30°, (c) 45°, (d) 60°, (e) 75°.

The interaction between rock size and rock bridge angle on the fluctuation coefficient of UCS can be analyzed from the perspective of rock mechanics. Based on the data analysis in Fig 8B, it is found that when the rock size is between 100 mm and 250 mm, the UCS fluctuation coefficient basically shows an increasing trend with the increase of the rock bridge angle. This is because within this size range, the internal defects and cracks of the rock are relatively obvious, and the increase in the inclination angle of the rock bridge leads to uneven stress distribution inside the rock, resulting in an increasing trend in the fluctuation coefficient of UCS. However, as the rock size gradually increases to 350 mm, the fluctuation coefficient of UCS gradually decreases, and the mechanical properties of the rock tend to stabilize, indicating that the influence of rock bridge angle on it gradually decreases.

### 3.3 Relationship between rock size and UCS

#### 3.3.1 Stress-strain curves analysis

According to the research content 2, Fig 8 gives the stress-strain curves of different rock sizes when the rock bridge angle is 15°, 30°, 45°, 60°, and 75°, respectively.

**Fig 9:**
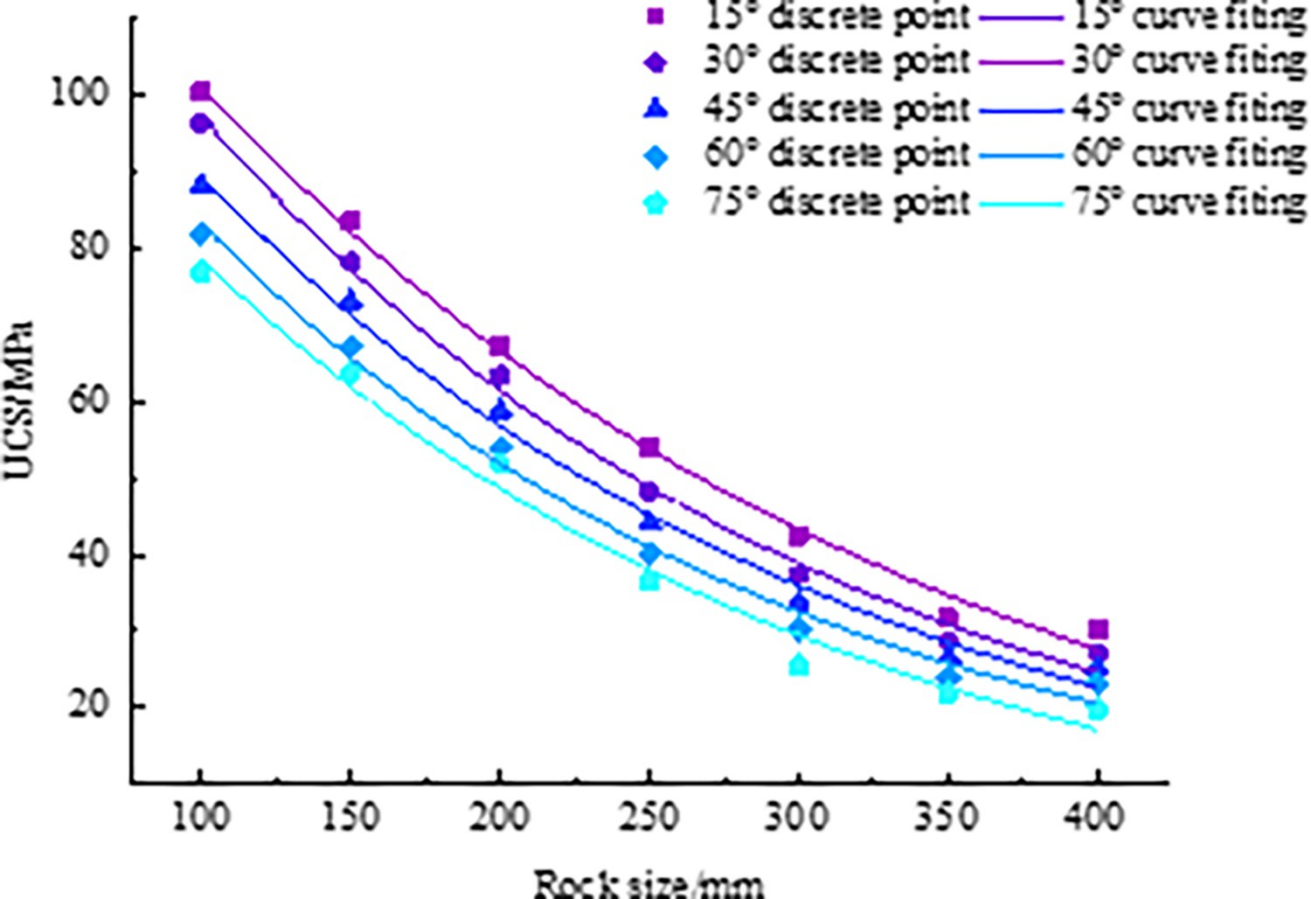
Fitting curves of UCS in different rock sizes.

According to Fig 9, the UCS under each scheme is obtained and summarized in Table 7.

**Table 7:** UCS under different rock size.

Simulation program	Rock bridge angle/°	UCS /MPa						
		Scheme 1	Scheme 2	Scheme 3	Scheme 4	Scheme 5	Scheme 6	Scheme 7
		100mm	150mm	200mm	250mm	300mm	350mm	400mm
Scheme 6	15	100.44	83.51	67.28	54.17	42.63	31.69	30.24
Scheme 7	30	96.43	78.27	63.46	48.24	37.44	28.66	27.14
Scheme 8	45	88.25	73.16	58.88	44.18	33.84	26.84	25.26
Scheme 9	60	82.08	67.29	54.03	40.22	30.34	23.91	23.25
Scheme 10	75	76.94	63.87	51.96	36.54	25.56	21.86	19.91

Fig 8 shows that the smaller the size, the greater the peak strength of the rock, and the steeper the stress-strain curve. We can draw the following conclusion. (1) When the stress is low, the curve is closer and gradually deviates as the stress increases. This deviation phenomenon can be attributed to the occurrence of micro deformations such as structural plane relaxation, cracking, and propagation during the process of stress increase, leading to additional plastic deformation. (2) As the sample size gradually increases, the probability of containing weak structural planes in the sample increases, making the sample more prone to failure, resulting in a decrease in the peak strength of the curve. (3) When the sample size is 350 mm larger, the stress-strain curve relationship is relatively close, indicating that the strength value of the rock tends to stabilize when a certain size threshold is reached.

To explore the influence of rock size on UCS, the analysis is combined with Fig 8C and Table 7. When the size of the rock increases from 100 mm to 400 mm, the corresponding UCS is 88.25 MPa, 73.16 MPa, 58.88 MPa, 44.18 MPa, 33.84 MPa, 26.84 MPa, and 25.26 MPa, respectively. The UCS decreases with the increase of rock size, indicating a negative correlation between rock size and UCS.

According to the data of Fig 9 and Table 7, it can be found that there is a certain correlation between the rock bridge angle and UCS. With the increase of the rock bridge angle, the UCS decreases gradually, and the peak and valley values appear at the rock size of 15° and 75°, respectively. Taking the rock size of 200 mm as an example, the UCS is 67.28 MPa when the rock bridge angle is 15°, and 51.96 MPa when the rock bridge angle is 75°, with a difference of 22.77%. This indicates that there is a negative correlation between the rock bridge angle and UCS.

Based on the analysis results, it can be concluded that there are negative correlations between rock size and rock bridge angle, and UCS.

#### 3.3.2 Curve fitting of rock size and UCS

The data in Table 7 are further visualized and the fitting curve of rock size and UCS is drawn, as shown in Fig 9. By analyzing the data and using statistical methods, the linear regression equations are obtained, as shown in Table 8.

**Table 8:** Fitting relationship between rock size and UCS.

Rock bridge angle/°	Fitting formula	Fitting coefficient (R^2^)
15	$\sigma (l)=7.14+153.24e^{0.0039l}$	0.99
30	$\sigma (l)=8.23+148.29e^{0.0041l}$	0.99
45	$\sigma (l)=9.27+143.12e^{0.0043l}$	0.99
60	$\sigma (l)=10.12+138.19e^{0.0045l}$	0.99
75	$\sigma (l)=11.22+133.28e^{0.0047l}$	0.98

where *σ*(*l*) is rock UCS, unit: MPa; *l* is rock size, unit: mm.

By analyzing Fig 9, it is found that the UCS decreases gradually with the increase of rock size. This trend is due to the presence of more defects or microcracks inside large-sized stones, which are more susceptible to stress damage than small-sized stones. When the size of the rock exceeds 350 mm, it is found that the decreasing trend of its UCS gradually stabilizes. This is because as the size of the rock increases, the density and size distribution of internal defects gradually stabilize, leading to a gradual decrease in the impact of defects on UCS. Therefore, the size effect has a significant impact on the mechanical properties of rocks, and it needs to be fully considered and analyzed in engineering practice.

The fitting formula in Table 8 shows that the appropriate degree between rock sizes and UCS are well, which can provide a simple and reliable method for solving the UCS in engineering practice.

#### 3.3.3 Mathematical model of rock size and UCS

According to Table 8, the relationship between rock size and UCS conforms to the exponential function, and the model is proposed as follows:

$$\sigma (l)=c+de^{fl}$$ (6)

Where *σ*(*l*) is the UCS, unit: MPa, *l* is the rock size, unit: mm, *c*, *d* and *f* are regression parameters, which can be obtained by linear regression.

**Fig 10:**
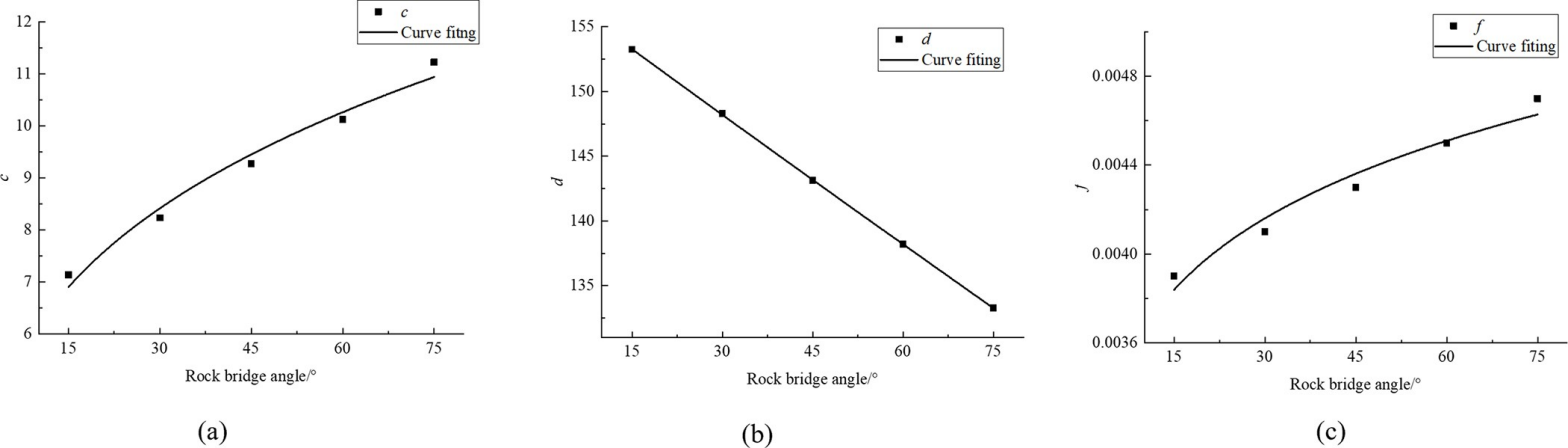
Fitting curve of parameter and rock bridge angle (a) c, (b) d, (c) e.

The regression parameters in Table 8 are sorted into Table 9. The regression parameters and the rock bridge angle are further fitted and plotted into charts, and the results are shown in Fig 10.

**Table 9:** Regression parameters under different rock bridge angles.

Parameter	Rock bridge angle				
	15°	30°	45°	60°	75°
*c*	7.14	8.23	9.27	10.12	11.22
*d*	153.24	148.29	143.12	138.19	133.28
*f*	0.0039	0.0041	0.0043	0.0045	0.0047

The fitting errors of Fig 10 are 0.97, 0.99, and 0.95. The relationships between parameters and rock bridge angle are:

$$c=3.82\alpha^{0.28}$$ (7)

$$d=-407.12+565.51\times 0.99^{\alpha }$$ (8)

$$f=0.0028\alpha^{0.12}$$ (9)

Substitute Formulas (7), (8), and (9) into Formula (6) to obtain the special relationship between rock size and UCS:

$$\sigma (l)=3.82\alpha^{0.28}+(-407.12+565.51\times 0.99^{\alpha })e^{0.0028\alpha^{0.12}l}$$ (10)

Eq (10) is suitable for estimating the UCS of rocks with different rock sizes. However, when measuring the size, it is necessary to maintain a fixed 2:1 side length ratio, which limits its applicability.

### 3.4 Relationship between rock bridge angle and CCS

The rock characteristic size is used to obtain the size required for stable rock mechanical parameters. In rock mechanics, the characteristic size plays a very important role in the study of rock mechanics characteristics, and its calculation method is provided in reference[24].

$$\left|k|=|\frac{de^{\left(-l/f\right)}}{f}\right|$$ (11)

|k|≤γ (12)

$$l\geq [\ln(\frac{d}{f})-\ln\gamma ]f$$ (13)

Where γ is the absolute value of the acceptable slope. It can be considered that $[\ln(\frac{d}{f})-\ln\gamma ]f$ is the solution formula of characteristic size.

#### 3.4.1 Theoretical model of characteristic UCS and rock bridge angle

**Fig 11:**
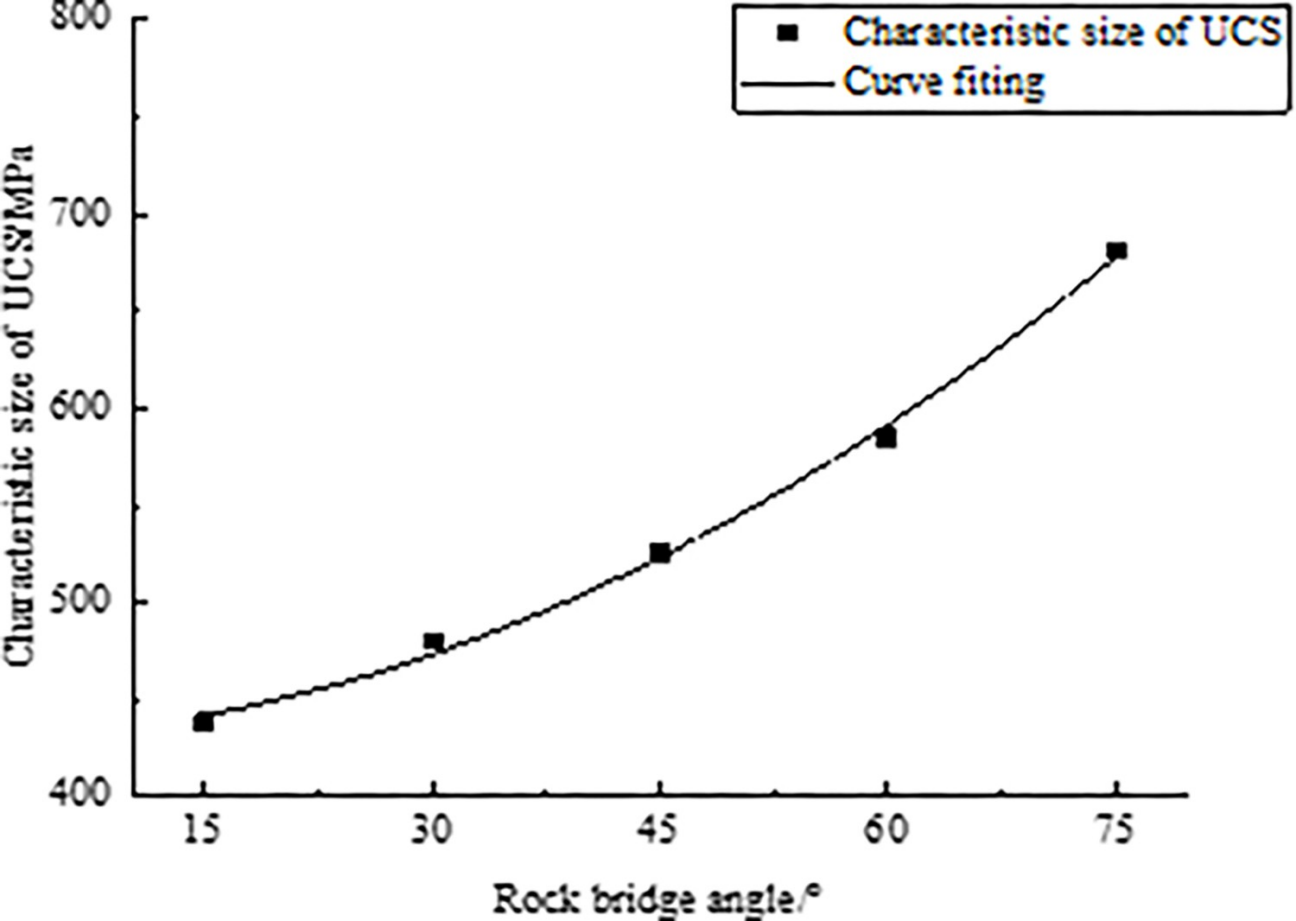
Fitting curve of characteristic size of UCS and rock bridge angle.

According to the Eq (13), the rock characteristic sizes under different rock bridge angles (15°, 30°, 45°, 60°, 75°) are calculated, and the calculated results are summarized in Table 10. Furthermore, in Fig 11, the relationship between the rock bridge angle and characteristic size is plotted to observe the trend and pattern of changes in the characteristic size of rocks under different rock bridge angle.

**Table 10:** Relationship between characteristic size and rock bridge angle.

Rock bridge angle/°	15	30	45	60	75
Characteristic size/mm	437.99	479.82	525.67	583.97	681.24

It can be concluded from Fig 11 that with the increase of rock bridge angle, the corresponding characteristic size of rock UCS increases gradually. To describe the trend more accurately and obtain the corresponding function, the collected data were fitted and analyzed. From the analysis results, it can be found that the relationship has a power function form. Therefore, the following power function relationship is established.

$$D(\alpha )=431.14-0.06\alpha^{1.92}$$ (14)

Where *D*(*α*) is the characteristic size, unit: mm; *α* is the rock bridge angle, unit:°.

Eq (14) can be used to infer the characteristic size of UCS by measuring the angle of rock bridges, but it is only applicable to rocks containing a set of rock bridges angles.

#### 3.4.2 Theoretical model of CCS and rock bridge angle

**Fig 12:**
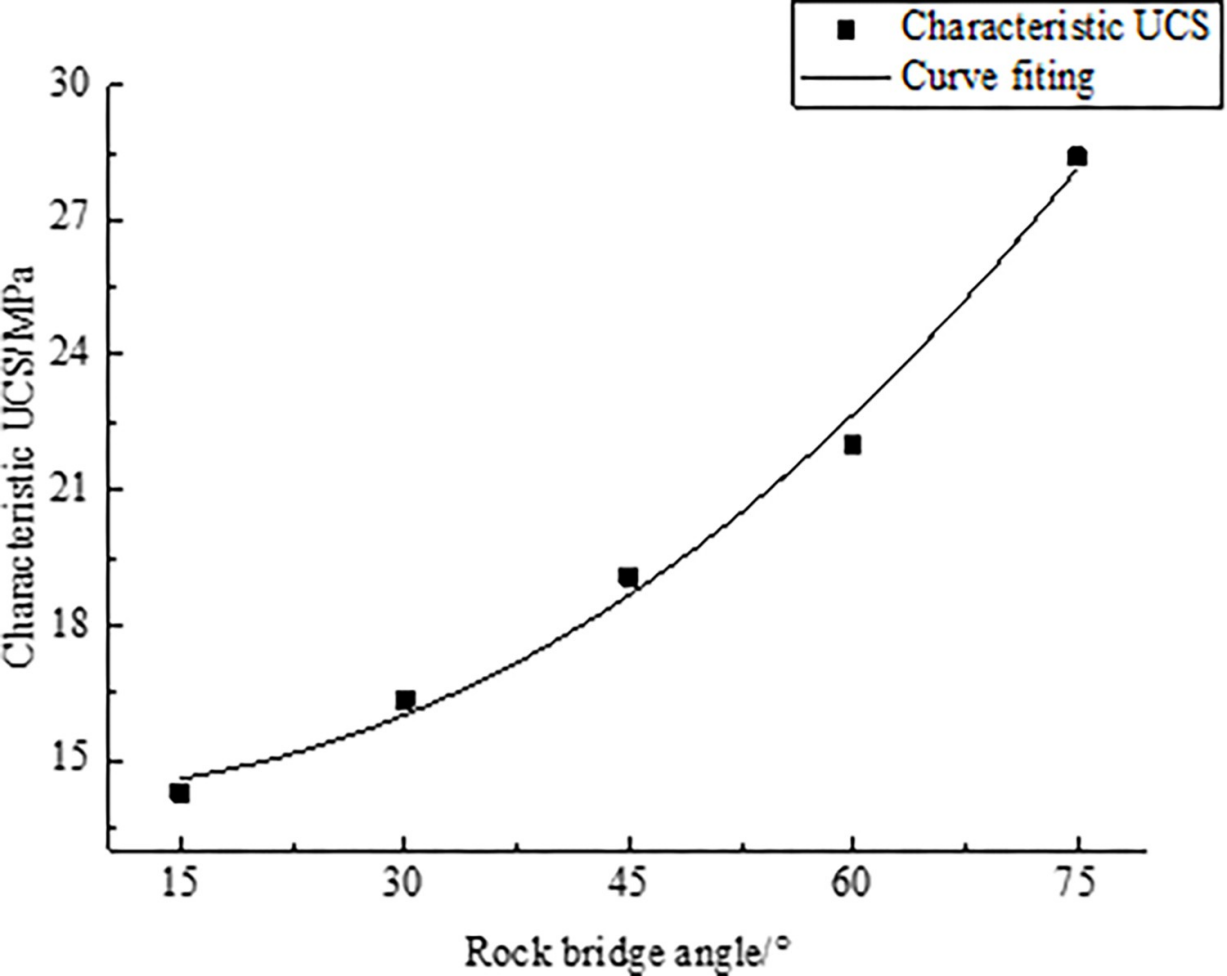
Fitting curve of CCS and rock bridge angle.

In order to calculate the characteristic compressive strength (CCS), the value of the characteristic size of rock UCS given in Table 10 are used and substituted into Eq (6) for calculation to obtain the corresponding characteristic compressive strength of rocks. The calculation results are summarized in Table 11 to facilitate the comparison and analysis of the characteristic compressive strength of different rock samples. At the same time, the linear regression analysis of the rock’s CCS and the rock bridge angle is carried out, and the fitting curve between them is obtained, as shown in Fig 12.

**Table 11:** Relationship between CCS and rock bridge angle.

Rock bridge angle/°	15	30	45	60	75
Characteristic UCS/MPa	14.28	16.34	19.07	22.01	28.41

According to Fig 12, as the rock bridge angle increases, the CCS gradually increases. A mathematical formula can describe this trend. According to the regression analysis results, the following regression formula is obtained:

$$\sigma_{w}(\alpha )=14.21-20.76\alpha^{2.23}$$ (15)

Where *σ*_*w*_(*α*) is the CCS, unit: MPa; *α* is the rock bridge angle, unit:°.

Eq (15) is used to derive the CCS of rocks with measured rock bridge angles, which is only applicable to rocks containing a set of rock bridge angles.

### 3.5 Validation analysis

To verify the accuracy of Eq (6), experimental data from reference[25] were cited (page 69, Figs 3 and 4) and summarized in Table 12.

**Table 12:** Relationship between UCS and rock bridge angle.

Rock bridge angle/°	30°	60°	90°
UCS/MPa	23.06	22.29	21.90

**Fig 13:**
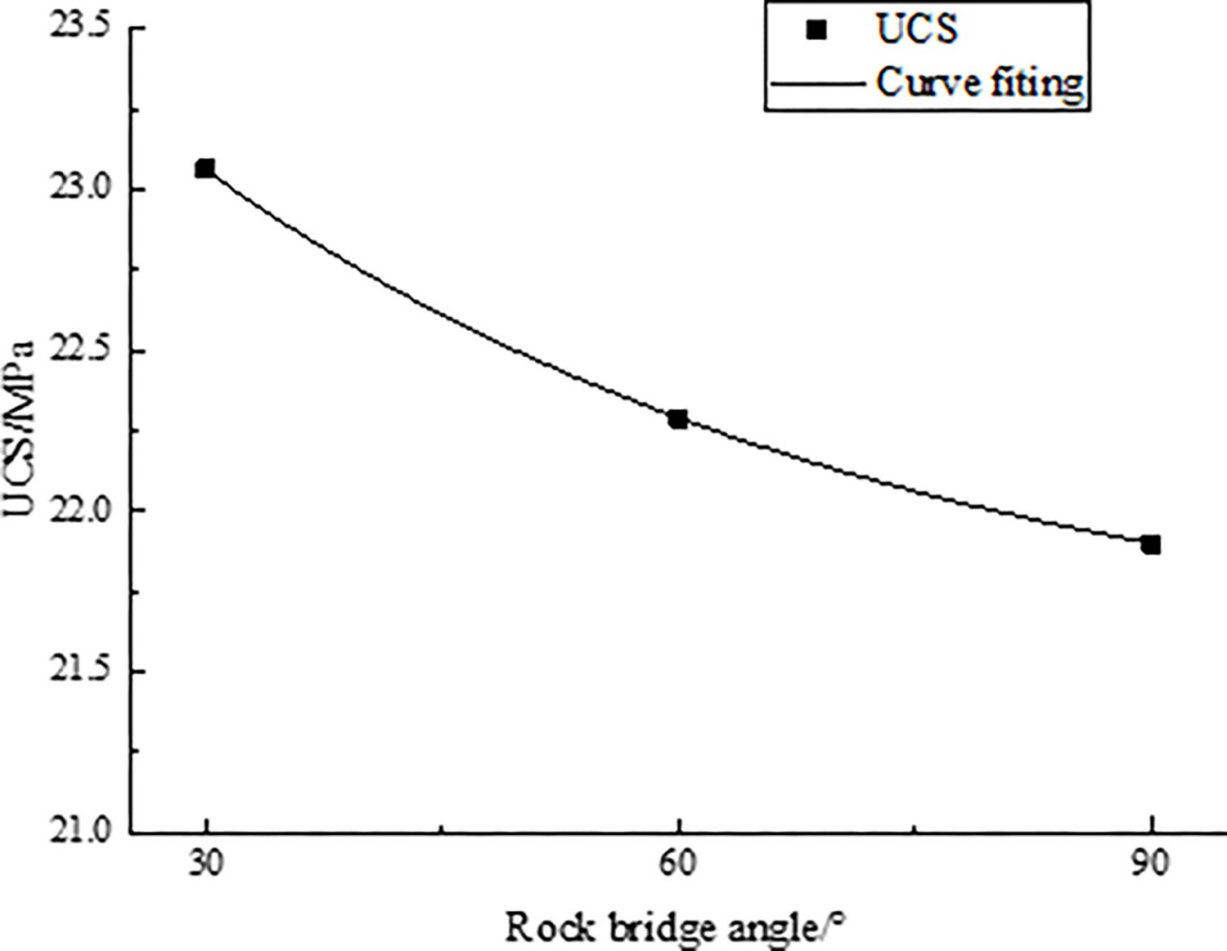
Curve fitting of rock bridge angle and UCS.

Import the data in Table 12 into Origin software for processing and analysis. By fitting the data, the fitting curve between the rock bridge angle and UCS is drawn, as shown in Fig 13.

Through the linear regression analysis of Fig 13, the relationship between the rock bridge angle and UCS is obtained as follows:

$$\sigma (\alpha )=21.49+3.08\alpha^{0.02}$$ (16)

The mathematical model proposed by Eqs (6) and (16) has a high degree of agreement, indicating that the results of numerical simulation are consistent with the indoor experiment, proving the accuracy of Eq (6).

## 4.Discussion

This paper establishes the following relationships: (1) rock bridge angle and UCS; (2) rock size and UCS; (3) CCS and rock bridge angle.

(1) The relationship between UCS and rock bridge angle is obtained by analyzing the law of UCS changing with the rock bridge angle. In existing research, scholars have rarely established mathematical models for the relationship between rock bridge angle and UCS. However, the Eq (4) obtained in this study can be used to calculate the UCS of rocks with rock bridge angle, which has important reference value for engineering practice.

(2) The relationship between UCS and rock size is obtained through linear regression. In existing research, scholars mainly study the effects of aspect ratio [11], water content [12], and strain rate [14] on UCS, but rarely establish mathematical models for UCS and rock size, and rarely consider the influence of rock bridge angle.

(3) The relationship between the CCS of rocks and rock bridge angle is also obtained by linear regression, the conclusions obtained can provide reference value for engineering practice.

However, this article only used numerical simulation as the research method and obtained the above formula based on it. The applicability of the formula is relatively small and has certain limitations. Therefore, to expand the applicability and credibility of the formula, future research should increase experimental controls and expand experimental subjects, including more rock bridge inclinations, sized rocks, and types of rocks, which will help improve the applicability of the formula.

## 5.Conclusion

To meet the evaluation requirements for the strength of rocks of different sizes in engineering sites, this study explores the variation patterns of UCS, fluctuation coefficient, and characteristic size of rocks. The results indicate that:

1) The UCS decreases with the increase of the rock bridge angle, showing a power function relationship.

2) The UCS decreases with the increase of rock size and tends to stabilize when the rock size is greater than 350 mm, showing a significant size effect.

3) The fluctuation coefficient of UCS is influenced by the rock bridge angle and rock size. It increases with the increase of the rock bridge angle and decreases with the increase of the rock size. It is less than 5% when the rock size is 350 mm.

4) The CCS and characteristic size are influenced by the rock bridge angle, and both increase with the increase of the rock bridge angle.

In the future, we will further explore these conclusions and apply them to more complex engineering to conveniently calculate the strength of rocks.
